# Oxidation Treatments Using Hydrogen Peroxide to Convert Paper-Grade Eucalyptus Kraft Pulp into Dissolving-Grade Pulp

**DOI:** 10.3390/molecules28237927

**Published:** 2023-12-04

**Authors:** Andrea Vera-Loor, Panagiota Rigou, Gérard Mortha, Nathalie Marlin

**Affiliations:** Laboratory of Pulp and Paper Science and Graphic Arts, University Grenoble Alpes, Centre National de la Recherche Scientifique (CNRS), Grenoble INP, LGP2, F-38000 Grenoble, France; panagiota.rigou@grenoble-inp.fr (P.R.); gerard.mortha@grenoble-inp.fr (G.M.); nathalie.marlin@grenoble-inp.fr (N.M.)

**Keywords:** dissolving pulp, hydrogen peroxide, periodate oxidation, paper grade pulp

## Abstract

Converting paper-grade bleached Kraft pulp into dissolving pulp using eco-friendly chemicals on-site at the mill is a challenge for the pulp industry. In this study, two oxidation systems are evaluated: the first one is based on the use of hydrogen peroxide at various levels of alkalinity; the second one investigates the use of sodium periodate followed by hydrogen peroxide to convert aldehydes into carboxyls and enhance their hemicelluloses removal. Our results have shown that when using only peroxide, the removal of hemicelluloses was not sufficient to improve the pulp’s dissolving ability. Conversely, the periodate–peroxide system proved to be more efficient. Results regarding the pulp purity, solubility, degradation (pulp viscosity and cellulose molecular mass distribution), brightness, and its potential applications were discussed.

## 1. Introduction

The main products obtained from dissolving pulps are used in textiles, tires, coatings, paints, and tobacco products, as well as in food and pharmaceutical products [1]. By the end of 2014, the pre-hydrolysis Kraft (PHK) process represented 56% of the world dissolving pulp production, whereas the acidic sulfite (AS) process represented 45%. A total of 88% PHK and 80% AS dissolving pulps were used for viscose production [2]. When using softwoods and hardwoods as raw materials, drastic conditions are needed to obtain a high-quality dissolving pulp, as residual impurities can adversely affect its final properties [3].

Producing dissolving cellulose is not a normal focus for conventional paper-grade Kraft pulps. It implies the breaking of the strong hydrogen bonds between the cellulose chains and replacing them via interactions with a solvent, or through derivatization of the cellulosic OH-groups to prevent H-bond formation [4]. Hemicelluloses are important components of paper-grade pulps, but a key issue is their selective removal for dissolving pulp applications. The purification process aimed towards producing dissolving pulps (R18 > 96%) involves both the removal of non-cellulosic materials, such as lignin and extractives, and the removal of hemicelluloses. Additionally, for some applications, changing the molecular weight distribution of the cellulose to a narrow, monomodal type with a minimum amount of low molecular weight fractions can be required, as it has been demonstrated that not doing so can negatively affect the further dissolving process behavior or significantly reduce the strength characteristics of the final products, thus lowering their final quality [3].

As an alternative to the PHK process (which requires heavy investments in a conventional Kraft mill), cold caustic extraction (CCE) can be conducted at room temperature after Kraft pulping to complete cellulose purification, removing an important portion of the residual hemicelluloses. As a drawback of CCE, some mercerization might happen and may compromise the pulp’s ability to dissolve and reactivity to certain chemicals. Mercerization is the conversion of cellulose I to cellulose II, leading to stronger inter- and intra-molecular bonding [5]. Another negative effect of strong alkaline treatment is cellulose fibril aggregation, caused by the removal of low-molecular weight cellulose and hemicelluloses [6].

Cellulose reactivity is a key parameter for dissolving-grade pulp, which is defined by the ability of the hydroxyl groups at C2, C3, and C6 to react with other chemicals [7]. A CCE post treatment may allow to increase such reactivity by decreasing the cellulose’s degree of polymerization or by increasing the surface area of the fibers. Dissolving pulp reactivity is a complex issue; for example, mercerized cellulose is resistant to acetylation under conventional conditions, while being reactive in the viscose process [8].

Consequently, the use of caustic extraction steps for the upgrading of paper-grade pulp has become of interest. For example, a modified CCE, at 84 mg/L NaOH and with a lower consistency than usual, allowed the improvement of a commercial bleached Kraft pulp through performing twelve CCE stages at 25 °C for 30 min each [9]. Furthermore, good dissolving-grade pulp properties have been observed when optimizing the extraction step during the bleaching sequence, with 60–80 g/L NaOH at 80 °C: optimized treatments allowed researchers to reach a high selectivity regarding xylan removal while the cellulose was preserved [10]. Two systems have been formerly studied by Arnoul-Jarriault et al. (2015) with bleached softwood Kraft pulp: a modified caustic extraction, allowing a 70% removal of hemicelluloses at temperatures above 90 °C; and a two-step operation, which consisted of an acidic stage (>150 °C) followed by a hot caustic extraction (HCE) at 110 °C, allowing hemicelluloses removal of more than 60%. The latter process led to strong cellulose degradation, yet it remained in the acceptable range for dissolving-grade pulps, in addition to the fact that no cellulose II was formed compared to the modified caustic extraction formerly mentioned [11].

In our study, conventional CCE was referred to as the “single E” treatment; it was used as a reference for comparison with the other treatments under study, involving the application of hydrogen peroxide (H₂O₂) and additives on conventional fully bleached Kraft pulps.

Hydrogen peroxide has been widely used in the pulp and paper industry since the 1980s. Nowadays, due to the versatility of hydrogen peroxide, it is used as reinforcement agent after oxygen delignification, and in alkaline extraction stages to avoid the yellowing induced by sodium hydroxide [12]. The conventional operating conditions of the P stage are presented in Table 1:

Nevertheless, H₂O₂ is easily decomposed. This decomposition is catalyzed by metal ions and alkali and forms intermediate oxidative free radicals that can degrade cellulose. To avoid this, chelating agents (Q stage), and sodium silicate are used in industrial production. Yet, some remaining parts of the metal ions in pulp can either oxidize or reduce H₂O₂ [13]. Under alkaline conditions, the superoxide radical anion (O_2_•–) can be generated, which can form hydroperoxide anion (HOO–) that can further undergo a metal-catalyzed disproportionation reaction forming hydroxyl radicals (HO•). Most of these reactions are extremely quick but others can go very slowly [3]. The decomposing products lead to the introduction of hydrophilic carboxyl groups in the pulp, the cleavage of different types of bonds, and the dissolution of lignin. 

Some studies have investigated the use of H₂O₂ with additives for purposes other than bleaching [14,15,16,17]. Walger et al. (2017) studied the hydrogen peroxide–copper phenanthroline system in neutral and alkaline pH to strip the colors of recycled fibers. However, when the color stripping was improved, the cellulose was also partially damaged [18]. The same system has been investigated by Vladut et al. (2011) for wood pulp delignification. Again, the efficiency was strongly dependent on the pH: pH > 9 was needed for delignification, but pH > 11 was preferable to achieve the maximum delignification [15].

The purpose of oxidizing carbohydrates via the periodate ion, as in the second strategy proposed in the present study, is to introduce dialdehyde groups into polysaccharides [19]. Periodate-oxidized cellulose is often referred to as dialdehyde cellulose (DAC). Moreover, DAC is biodegradable, biocompatible, and its aldehyde compound can be further converted into carboxylic groups, primary alcohols, or amines, which can serve as useful intermediates for cellulose-based specialty materials [20]. Some other recent developments have also been presented, like crosslinking reactions and further modifications of nanocelluloses, for applications in paper wet strength additives [21] or bio-based adhesives produced from microcrystalline cellulose [22].

**Figure 1:**
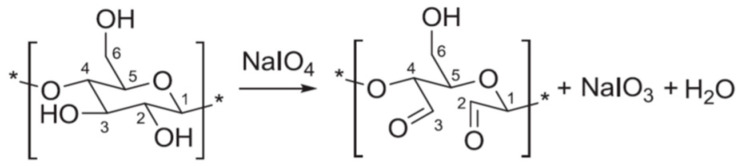
Periodate oxidation of cellulose.

It is well known that periodate (IO_4_^−^) is a unique reagent that can selectively oxidize the vicinal hydroxyl groups at carbon 2 and 3 in an anhydroglucose unit (AGU) into two aldehyde groups, simultaneously breaking the C–C bond. This reaction is illustrated in Figure 1. Peroxide oxidation is accompanied by a decrease in crystallinity and uneven distribution of the aldehydes [23], because of the limited cellulose chain accessibility in fibrils.

As a side reaction, cellulose depolymerization takes place; the higher the aldehyde content, the more the degradation [24]. However, breaking the longest chains of cellulose could be, in the case of this study, a desired effect since it might aid the conversion of Kraft pulps into dissolving pulps. Moreover, since hemicelluloses are more accessible in the fiber cell wall than cellulose fibrils, cutting the short chains of hemicelluloses could also help their removal, which possibly might occur simultaneously as the removal of the short, degraded cellulose chains. However, it should be kept in mind that for some dissolving pulp applications, the pulp viscosity can be lowered but not too much, like in the viscose process for instance, where it is generally kept within a range of 200–300 mL/g.

The aim of this study was to evaluate the use of H₂O₂ to convert paper-grade pulp into dissolving-grade pulp through applying unconventional combinations of treatments using chemicals commonly found (or applicable, in the case of periodate; indeed, despite its high cost, it can be fully recycled from iodate using ozone [25]) in paper mills.

Two systems have been studied:

- The first one is based on the combination of hydrogen peroxide oxidation with alkaline extraction of hemicelluloses, referring to the well-known HCE or CCE treatments. The idea is to combine two effects: hemicelluloses extraction and cellulose oxidation. The hemicelluloses extraction should favor the dissolution of the pulp, and the oxidation should allow for the introduction of hydrophilic groups on the cellulose backbone, improving the reactivity.
- The second system investigates the use of solely oxidative agents: sodium periodate and hydrogen peroxide. The purpose is, firstly, to introduce some carbonyl groups into the cellulose via periodate oxidation of the alcohols, followed by over- oxidation of the carbonyls into carboxyl groups, since it was observed that hydrogen peroxide alone did not enrich the pulp with enough carboxyl groups to favor substrate dissolution in a significant manner.

## 2. Materials and Methods

Two pulps were selected: the ECF fully bleached hardwood Kraft pulp, from Portugal, referred to as HW (intrinsic viscosity (IV) in CuED of 496 mL/g, DPv of 692), and the ECF fully bleached softwood Kraft pulp from Finland, referred to as SW (IV in CuED of 745, DPv of 1086). 

### 2.1. Alkaline and Peroxide Treatment

A combination of conditions between CCE and HCE have been selected for the treatment of 10 g of cellulosic substrate: including a temperature of 75 °C, as this is in the lower range for HCE, and at the same time, is still in the range of a P stage. The NaOH concentration should be carefully chosen: not too high to avoid the conversion of cellulose I into cellulose II, and not too low, at the risk of limiting the hemicelluloses extraction. We selected 75 g/L NaOH, as in a conventional CCE; yet the pulp consistency was set at 10%, as is usual in the conventional P stage for bleaching and in HCE treatment. Moreover, a high dose of H_2_O_2_ was applied, i.e., 10%, to compensate for the high alkaline charge.

To study the effect of the EP treatment, P, E, and E + P treatments, with intermediate washing after E, were carried out as controls. These conditions are gathered in Table 2. After applying the above-described treatments, characterization tests were performed, and the results are discussed in the following section.

### 2.2. Periodate Oxidation

A total of 20 g (oven-dried weight basis) of cellulosic substrate was homogenized in a Lhomargy disintegrator for 1 min, and later filtrated on a sintered glass crucible of porosity no. 2 to remove the excess water. The substrate was then transferred into a 2 L Erlenmeyer flask and filled with 1 L of NaIO_4_ titrated solution; water was then added to reach a weight of 1250 g and 1.6% pulp consistency. Different amounts of NaIO_4_ were tested: 54%, 27%, and 13.5% (% expressed as weight of NaIO_4_/weight substrate). The reaction took place in dark conditions and was left stirring for 2 h at room temperature. After the reaction, the suspension was filtered and washed with deionized water; however, this was not enough to remove the acidic medium. For this reason, after a couple of washes with water and filtration, the suspension was soaked for 10 min in 100 mL of a 20 g/L NaHCO_3_ solution, before filtering again and continuing the washing with deionized water until reaching a neutral pH. The washed and filtrated substrate was divided into two parts of same weight: one part to use in the characterization for evaluating the effect of the periodate oxidation, and the second part to start the next oxidation with H_2_O_2_ (P stage), as described below.

### 2.3. P Stage after Periodate Oxidation

The overoxidation with hydrogen peroxide after the periodate oxidation was performed in mild conditions with a low dosage of hydrogen peroxide with slight alkalinity. The H_2_O_2_ dose was 2% and the NaOH was set at 1.2% or 1.5%, depending on the substrate (weight % based on initial cellulosic substrate before periodate oxidation). The other operating conditions were 75 °C, 1 h of reaction time, and 10% pulp consistency. After the second oxidation, the H_2_O_2_ consumption was monitored using the effluent at the end of the reaction via iodometry.

### 2.4. Characterization

The pH was measured at the beginning of the reaction, after the chemical addition, and at the end of it, before washing. The consumption of H_2_O_2_ was determined immediately after each oxidation trial, and this was measured via iodometric titration in the presence of sulfuric acid. The titration chemical was sodium thiosulfate (0.2 M); the brightness (%ISO) was determined according to the standard ISO 2470-1:2016 method; the alkali resistance of the pulp (commonly addressed as R18) was calculated following the standard method ISO 699-1982, which is applicable only for bleached and delignified pulps; the carboxyl group quantification was estimated following the standard method TAPPI T237 cm-08; the average viscosity degree of the polymerization was estimated following the standard method ISO 5351:2010; and, finally, the dissolution in the NaOH:urea:water system, as well as the cellulose tricarbanilation trials monitored with DLS, followed by the SEC analyses, were conducted following the protocols presented by Vera-Loor et al. (2023) for evaluating the dissolving ability of cellulosic pulps [26].

## 3. Results

### 3.1. H_2_O_2_ Consumption and Intrinsic Viscosity

**Figure 2:**
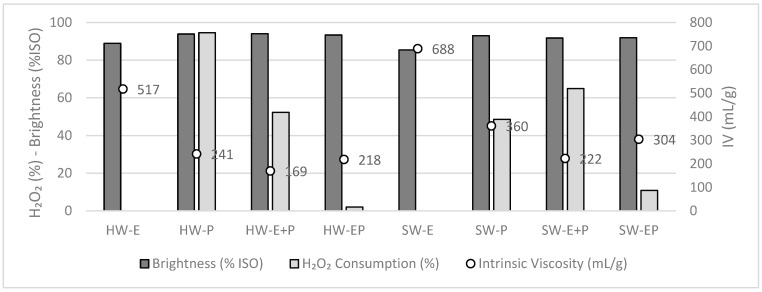
H_2_O_2_ consumption, brightness (%ISO), and intrinsic viscosity (mL/g) after oxidation with 10% NaOH (HW-IV_initial_, 496 mL/g and SW-IV_initial_, 745 mL/g).

The consumption of H_2_O_2_ was monitored, as well as the final CuED viscosity (IV value) and brightness (%ISO), and these results are presented in Figure 2. For all cases, the final pH was higher than 11.

As a general observation, for both substrates and all treatments, even those with a high alkalinity (10% NaOH), no detrimental effect on the brightness (%ISO) was observed. 

For both SW and HW, the use of the extraction alone (E) did not strongly affect the intrinsic viscosity; for HW it went from 496 mL/g before E to 517 mL/g after E (thus, a slight increase rather than a decrease), and for SW it went from 745 mL/g to 688 mL/g. The E stage alone affects mostly the hemicelluloses and the short chains of cellulose, which are a minority in the cellulosic pulps, explaining the low effect of the E stage.

When looking at the results of the single P stage (10% H_2_O_2_, and 2% NaOH), it can be observed that the intrinsic viscosity is highly affected for both samples. H_2_O_2_ at high dosage in alkaline conditions is known to depolymerize cellulose, as observed here. In the case of the E + P treatment, a stronger cellulose depolymerization occurs, even more pronounced than that with P only. This could be explained by the fact that after E, less hemicelluloses are present; thus, in P after E, H_2_O_2_ mainly reacts with cellulose, which is much more affected. Finally, after EP (no intermediate wash included), the intrinsic viscosity values are decreased due to the high dosage of H_2_O_2_. It can be noted that the chosen conditions did not allow the total consumption of H_2_O_2_ in all of the trials containing H_2_O_2_, even in the case of EP, where both the H_2_O_2_ and alkali doses are high. The high quantity of H_2_O_2_ applied is thus responsible for the strong cellulose degradation. To target intrinsic viscosity values around 400 mL/g for dissolving pulp applications, the H_2_O_2_ dose should be lowered.

**Figure 3:**
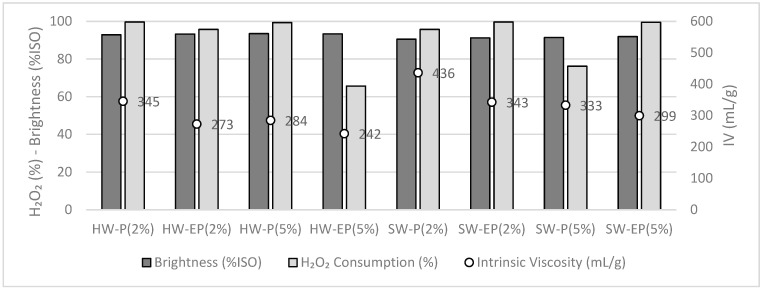
H_2_O_2_ consumption, brightness (%ISO), and intrinsic viscosity (mL/g) after oxidation with 2% and 5% NaOH (HW-IV_initial_, 496 mL/g and SW-IV_initial_, 745 mL/g).

For these reasons, in the following trials, the P and EP treatments were tested using lower H_2_O_2_ doses, from 10% to 2 and 5%. The other conditions given in Table 2 were maintained. The effect of this on the pulp brightness, intrinsic viscosity, and H_2_O_2_ consumption are given in Figure 3.

In these new trials, with a reduced H_2_O_2_ input and maintaining high alkalinity in EP, the H_2_O_2_ consumption is higher and the cellulose depolymerization is lessened, but the intrinsic viscosity values are still rather low for viscose applications. For both substrates, the more interesting overall results in terms of brightness (%ISO) and chemical consumption were observed when using only the 2% H_2_O_2_. In the case of 5% H_2_O_2_, the oxidant consumption is less because the alkali dose, i.e., 2% NaOH, is too low. In the latter case, the cellulose oxidation is probably poor. EP again leads to more cellulose depolymerization than P alone; but this difference has become rather small. Regarding H_2_O_2_ consumption, it seems to be slightly less at higher alkalinity levels for the EP stage than in the P stage. The reason for this is not clear, especially since an opposite trend is observed in the case of SW. It is worth noting that when high charges of H_2_O_2_ were applied, the iodometric dosage of the effluent at the end of the P stage (to titrate the quantity of H_2_O_2_ consumed) could prove to be imprecise. Indeed, the effluent is enriched in dissolved organic matter, and the presence of dissolved carbonyl compounds is likely to interfere with the iodometric dosage of the oxidant.

Globally, HW and SW reached their highest intrinsic viscosities at low H_2_O_2_ dosages and when no strong extraction step was included. Brightness values were kept around 90%ISO, and almost a full consumption of H_2_O_2_ was evidenced.

Other parameters were monitored to evaluate their potential interest for dissolving pulps, such as MWD profiles, solubility, and purity. All of these are discussed below.

### 3.2. Alkali Resistance and COOH Quantification

To investigate the effects of the above treatments on hemicelluloses removal, R18 analyses have been performed on the pulps treated with 2% and 5% H_2_O_2_. The single P stage with low alkalinity (2%) has been included for comparison. The results are presented in Table 3.

Table 3 shows the R18 value for HW-REF as 90.6% before any treatment. After P alone (2% or 5% H_2_O_2_), cellulose purity did not improve significantly (91.3 and 90.7%, respectively). Thus, a single oxidation with hydrogen peroxide seems to slightly remove hemicelluloses, but this effect is not sufficient to achieve a removal of >95%. However, when looking at the combined EP treatment, the R18 increased to 96.9% for HW-EP (2%) and 95.3% for HW-EP (5%). Thus, more than 60% of the initial hemicelluloses were removed with EP, which reaches the general target for dissolving-grade pulp. However, it appears that EP performs less well than E alone, where the R18 values reached 99.1%. Therefore, the addition of H_2_O_2_ in the E stage has a negative effect on hemicelluloses extraction. Globally, to explain the above effects, the following assumption can be proposed: HW is a rather accessible substrate containing xylan, which can become a solution in a single E stage (with very strong alkalinity). Introducing H_2_O_2_ in this strongly alkaline medium leads to not only hemicelluloses extraction, but also polysaccharides oxidation and depolymerization. Some oxidized/depolymerized chains in the substrate can dissolve in the R18 test, leading to apparently less pure cellulose compared to E. Contrary to E and EP, P alone (at a low alkalinity) does not have the capacity to dissolve the hemicelluloses, thus leading to unchanged R18 values after oxidation.

For SW, the dissolution pattern exhibited similar tendencies, with R18 values ranking in the order E > EP > P > REF. The difference with HW is that SW has a lower initial purity (R18 of 79.7% for the SW-REF sample). P alone removes some hemicelluloses, but the dissolving pulp target is not reached. Yet, P removed hemicelluloses in the SW substrate, compared to HW, where no changes were observed. Differences in their hemicelluloses composition are perhaps the reason for this: the glucomannans of softwoods may be more sensitive to oxidation and depolymerization than the xylans of hardwoods. The EP treatment in SW removed more hemicelluloses than P, but the R18 remains too low. E was more efficient than EP, leading to the highest R18 value, above 92%, which is at a very low limit of the target range required for dissolving-grade pulps. It should be highlighted that a cellulose content above 95% is the general target for high-quality dissolving-grade pulps, yet 90% is also acceptable for some specialty pulps [1].

To better understand the actions of P and EP on the fully bleached pulp, the carboxylic groups of the substrates have been quantified, and these results are also included in Table 3. No changes were observed for SW compared to the initial pulp (SW-REF), regardless of the treatment applied, P or EP. In the tested conditions, H_2_O_2_ was not able to insert COOH groups into the substrate. For HW, the alkaline P treatment slightly oxidized the pulp, but when the extraction step E was included, this effect was reduced, which may likely be due to the dissolution of the carboxylated fragments. In any case, the extent of the oxidation is poor compared to what was expected. Such rates of oxidations may not be sufficient to significantly improve the dissolving ability of the studied substrates.

It is well known that polysaccharide oxidation using hydrogen peroxide in alkaline media generates carbonyl groups as a first step, and aldehydes and ketones as part of them being further oxidized into carboxyl groups in a subsequent step. However, it is observed here that these oxidizing conditions do not lead to carboxylation of the matrix. On one hand, as stated above, this might be explained by the dissolution of the carboxylated fragments. But on the other hand, the carbonylated cellulose encounters severe depolymerization in alkaline media due to β-alkoxy degradation, a fast reaction that can compete with carboxylation. Both phenomena are likely to contribute to the observed results in the strongly alkaline medium. Indeed, the β-alkoxy degradation reaction consumes hydroxide ions. Overall, both effects contribute to the observed inability of hydrogen peroxide to carboxylate the matrix.

### 3.3. Hydrogen Peroxide with a Previous Periodate Oxidation Stage

Unlike peroxide, which is known to generate both ketones and aldehydes on the cellulosic chains, aldehyde formation can be selectively obtained via periodate oxidation. The latter was applied first as an oxidative stage to convert the groups of vicinal alcohol in C2 and C3 into di-aldehydes. This was performed in conventional conditions, i.e., at a slightly acidic pH and room temperature (see Materials and Methods section) and with a large amount, between 13.5% and 54%, of periodate on the pulp (*w*/*w*). The periodate oxidation was followed by a classical P stage at a moderate alkalinity to over-oxidize the DAC into di-carboxyl cellulose (DCC). More interesting conditions are yet to be identified for potential future applications of dissolving-grade pulps, as a strong cellulose degradation is expected (but should be avoided) after these two steps of oxidation. For thise discussion, the periodate oxidation will be referred to as the first oxidation stage, and the hydrogen peroxide oxidation will be called the second oxidation. A cotton linters (CL) substrate has been included in the present section to observe the effect of the proposed treatments on a substrate free of hemicelluloses, CL-IV_initia_l, at 695 mL/g.

**Figure 4:**
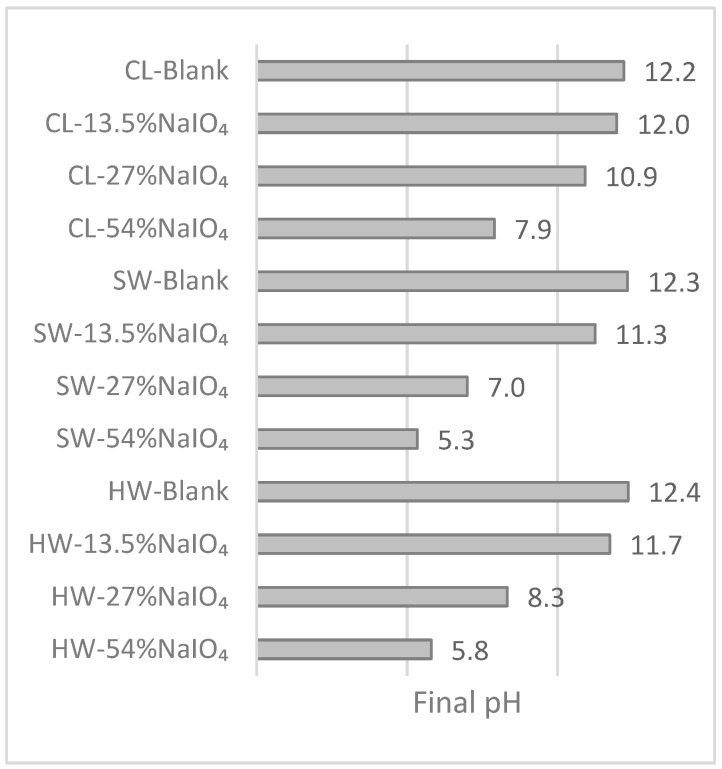
Final pH values after the substrate oxidation using a combination of NaIO_4_ followed by H_2_O_2_ in alkaline medium.

Figure 4 presents the final pH after the second oxidation step, i.e., after the P stage. 

A blank control test was also carried out. The blank test consisted of the same two-step oxidation procedure but without the NaIO_4_ addition in the first step. In this case, the pulp was placed into water at room temperature in dark conditions for 2 h. After this, the washing steps were reproduced as if a normal periodate oxidation had taken place, with the 10 min soaking in NaHCO_3_. Later, the second oxidation was performed with no further modifications. The results in Figure 4 show that the blank trials were alkaline, as expected, with pH values of 12.4, 12.3, and 12.2 for the HW, SW, and CL substrates, respectively.

In the presence of pulp, a decrease in the final pH after the two oxidations was observed; this was acidic (below 6) for SW and HW when using the highest dose of NaIO_4_, and close to neutral for CL. Even if bicarbonate was used to neutralize the acidic effluent of the periodate oxidation, the second oxidation required more alkali to reach an alkaline pH at the end. As a general observation, the lower the dose of NaIO_4_, the higher the final pH after the second oxidation. It is important to highlight that, as discussed in the previous section, the target final pH is alkaline, with the aim of reaching bleaching effluents similar to those observed in actual Kraft mills after the P stage. An alkaline effluent after the second oxidation was obtained when using 27% of NaIO_4_ and 13.5% NaIO_4_, enabling us to reach a final pH, like in a P stage, of between 11.3 and 12, as presented in Figure 4.

### 3.4. Intrinsic Viscosity, Brightness, and Peroxide Consumption

As explained above, the NaIO_4_ oxidation step prior to H_2_O_2_ oxidation aimed the insertion of dialdehyde groups during the first oxidation, followed by their conversion into di-carboxyls during the second oxidation. However, H_2_O_2_ oxidation may not have converted all of the carbonyl groups, and the cellulosic pulps may still contain carbonyls sensitive to β-alkoxy degradation under alkaline conditions. The consequence of this is that during the intrinsic viscosity analysis in CuED, the cellulose is depolymerized, resulting in false values for the DPv and intrinsic viscosity. To ensure the elimination of all carbonyl groups prior to DPv analysis, sodium borohydride (NaBH_4_) reduction was carried out after the second oxidation.

**Figure 5:**
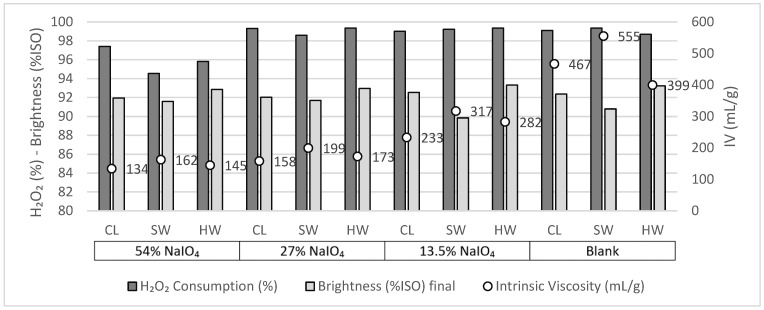
Intrinsic viscosity (mL/g), brightness (%ISO), and H_2_O_2_ consumption (%) of CL, SW, and HW after the second oxidation (CL-IV_initia_l, 695 mL/g; HW-IV_initial_, 496 mL/g; and SW-IV_initial_, 745 mL/g).

The intrinsic viscosity after the reduction with NaBH_4_, as well as the brightness and H_2_O_2_ consumption results of the substrates, all after the second oxidation, are presented in Figure 5.

For the three samples, regardless of the treatment applied, full H_2_O_2_ consumption was achieved (above 95%). Therefore, the peroxide consumption was not affected by the periodate oxidation conditions after washing. The brightness values were all around or above 90%ISO. The main effect observed concerned cellulose degradation, i.e., the decrease in the intrinsic viscosity in CuED.

Regarding the blank trials, they all presented a decreased intrinsic viscosity (IV). This was more accentuated when the sample exhibited higher initial intrinsic viscosity values: CL and SW, with initial IV values of 695 and 745 mL/g, respectively, were more affected by the oxidations than HW, with an initial IV of 496 mL/g. Yet, all of the blank trials exhibited a sufficient IV for dissolving-grade pulps, i.e., higher than 400 mL/g. 

When including a NaIO_4_ oxidation step prior to H_2_O_2_ oxidation, a stronger degradation was observed compared to the blank trials. The higher the dose of NaIO_4_, the more the decrease in the IV value. Using 54% and 27% NaIO_4_ led to IV values lower than 200 mL/g after the second oxidation, which is a too-low value for viscose application. A reduced dose of 13.5% NaIO_4_ led to an IV of 317 mL/g for SW, not so far from the objective. HW was the most affected, with a final IV of 282 mL/g, probably because the IV of its reference is the lowest one. Regardless of the initial IV before oxidation, the most affected substrate was CL, with a final IV of 233 mL/g. The absence of hemicelluloses in this substrate makes the cellulose more reactive during oxidation, thus the most damaged.

### 3.5. Carboxyl Quantification and R18 Test

Table 4 presents the quantification of the carboxyl groups after the second oxidation. No carboxyls were generated without periodate (the blank control trials). When periodate oxidation was performed with 13.5% NaIO_4_, some carboxyls were introduced but in very low amounts: for CL, these values increased from 3.8 to 4.7 meq/100 g of dry pulp; SW did not show relevant changes; and for HW, it increased from 6.3 to 7.9 meq/100 g, this being the highest increase. To explain these differences between HW and SW, it can be hypothesized that the oxidized mannans in SW may have dissolved more easily than the oxidized xylans in HW. In all cases, the alkaline medium during the P stage offered very good conditions for the dissolution of the carboxylated moieties of polysaccharides, which probably explains why the carboxyl amounts left in all substrates were quite small. Normal carboxyl contents for dissolving-grade pulps are in the range of 3 meq/100 g [3]. Even though our treatments did not significantly increase the presence of carboxyl groups, this is not a condition that needs to be fulfilled for dissolving-grade pulps.

The R18 test was also performed to estimate the purity of the cellulose; the results obtained with the lower dose of NaIO_4_ (13.5%), the blank, and the reference values are also presented in Table 4.

In general, a high cellulose content (R18) is required for dissolving-grade pulps. Lower grades exhibit a cellulose content of approximately 90%, while medium and high grades have a cellulose content of 94 to 95% and greater than 96%, respectively [1]. The low and medium grades are used for textile and cellophane production, while the high grades are mainly used for cellulose acetate and other specialty products.

Considering the purity required for dissolving-grades pulps, only the three CL samples and the HW-blank pulp offered acceptable values. The two other HW pulps (REF and NaIO_4_-oxidised) achieved the minimum limit of acceptability, whereas none of the SW substrates reached the range of a high-quality dissolving pulp (R18 > 95%). Typical variations can be observed which reflect that the R18 test not only eliminated hemicelluloses in these oxidized substrates, but also dissolved part of the oxidized fragments of the cellulose. For the case of the blank tests (no use of NaIO_4_), the accessibility was better in HW than in SW. When the periodate oxidation was carried out, some polysaccharides were oxidized and were removed during the R18 test, thus producing lower R18 values. This effect was more pronounced in the HW substrate, probably because of its better accessibility and easier dissolution of oxidized polysaccharide chains during the R18 test. This effect can also be related to the depolymerization induced by NaIO_4_, as the blank trials were much less degraded compared to the NaIO_4_ oxidations. This is also shown in the results discussed in the following section.

### 3.6. Dissolving Ability—E, P, and EP Treatments

**Figure 6:**
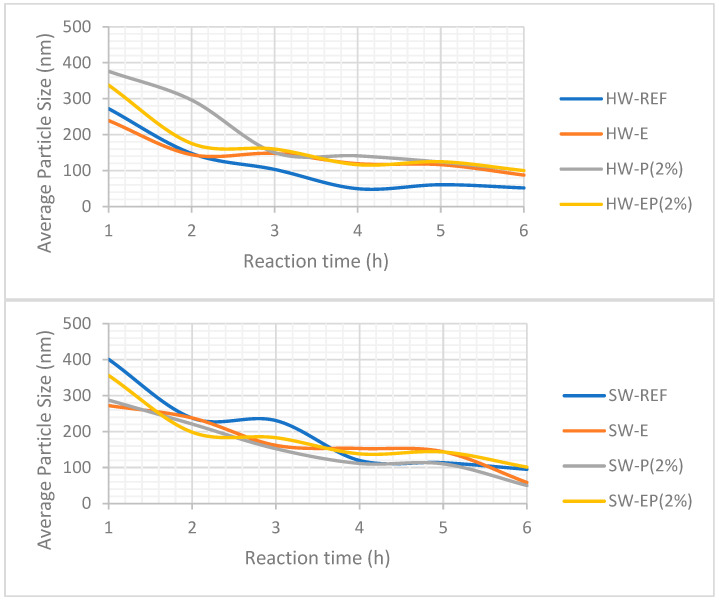
Dissolution during carbanilation of HW and SW treated with E, P, and EP (2% H_2_O_2_).

Dissolving ability has been assessed using the method described by Vera-Loor et al. (2023). This method monitors the dissolution ability with DLS during cellulose derivatization in DMSO (carbanilation via phenyl isocyanate), and, in addition, after the full reaction, it enables us to study the molecular weight distribution (MWD) of the cellulose, which is expected to be uniform for dissolving-pulp applications. Figure 6 presents the DLS information gathered during the carbanilation reaction in DMSO.

For HW, identified as the most accessible pulp in the previous section, the reference sample dissolved the fastest compared to all of the treated HW pulps (E, P, and EP). Additionally, when a caustic extraction step was included, i.e. HW-E and HW-EP (2%), the particle size decrease was rapid during the first two hours, and later the dissolution slowed down. This is not the case of the P (2%)-treated pulp, where a more regular evolution of the particle size was observed. One explanation for this might be that with caustic extraction, part of cellulose I is converted into cellulose II, and cellulose I dissolves during the first two hours of the reaction, while cellulose II dissolves more slowly. Anyhow, the dissolving behavior of HW-P (2%) was not better than the reference pulp, meaning that the partial oxidation of the substrate did not modify its dissolving ability in an organic reaction system (carbanilation in DMSO). 

SW pulp behaved differently: all of these dissolution curves presented very similar behavior. It can be concluded that its dissolving ability was not modified compared to that of the reference pulp, regardless of the treatment applied. 

HW and SW did not behave similarly: this may be due to the types of the hemicelluloses and their ability to easily react and dissolve in the carbanilation system, but it is also likely related to the structure of the SW substrate, which is much less accessible and less reactive than that of the HW, as previously discussed.

**Figure 7:**
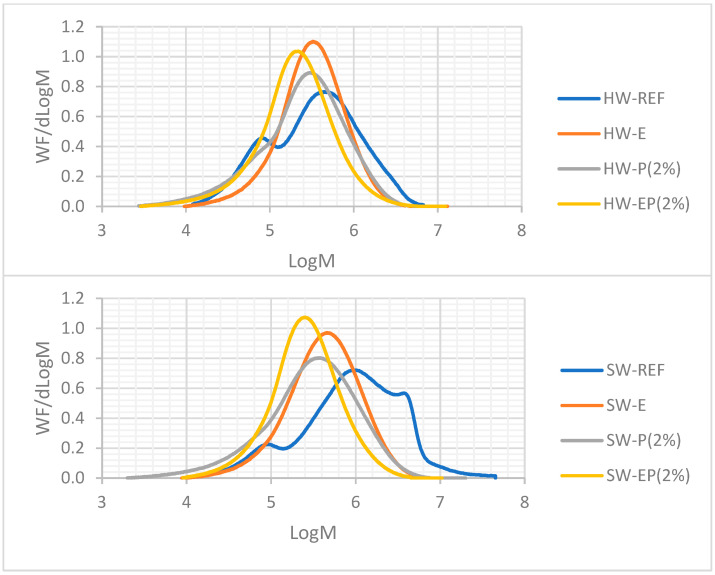
MWD of SW and HW treated with E, P, and EP (2% H_2_O_2_).

When observing the MWD profiles obtained via HPSEC analyses in Figure 7, it can be noted that for HW, a too-high dose of NaOH during the E stage was probably applied to extract the hemicelluloses, since almost all hemicelluloses were removed after E and EP. Additionally, it is assumed that part of cellulose I was converted into cellulose II, limiting the dissolution in DMSO. For SW, which contains more hemicelluloses, the NaOH dose was possibly insufficient to remove all hemicelluloses. Cellulose I was not significantly converted into cellulose II and the soda just acted as a hemicelluloses carrier. In SW, the reagents’ reactions improved the substrate accessibility, even when only P was applied, at a low alkalinity. Contrary to HW, the better accessibility of SW is probably a main factor that contributes to its dissolution, as it is more readily accessible and easily dissolved without the need of chemical agents. Untreated HW (REF) presented a bimodal profile, typical of the presence of cellulose and hemicelluloses. The MWD profile of HW-P (2%) was shifted toward a slightly lower mass, but the characteristic peak of hemicelluloses was still present. This indicates that P at a low alkalinity, even with a high peroxide charge, partially depolymerizes the cellulose but does not extract hemicelluloses (mostly xylans, in this case).

Complementary information obtained via HPSEC is presented in Table 5. During P (2%), the DPw decreased from 1215 to 814, but the dispersity, as an indication of the narrowness of the profile, remained unchanged (4.1 before and after P treatment). Using treatments with a strong alkalinity, E and EP, the hemicelluloses signal vanished (Figure 7), and the dispersity (Mw/Mn in Table 5) was reduced to two and three, respectively. For SW, the same trends were observed. The untreated SW exhibited a bimodal profile with both cellulose and hemicelluloses fractions, but with an additional shoulder around log M = 6.5, which typically indicates aggregation. For SW-P (2%), the cellulose signal was shifted towards a lower mass without hemicelluloses removal, as in the HW case. E and EP treatments in SW were able to remove hemicelluloses since the signal disappeared, with a significant reduction of the dispersity, indicating a much better uniformity of the MWD profile. A lower dispersity was observed for SW when H_2_O_2_ was added in the caustic extraction stage; here, H_2_O_2_ addition is positive, except for the strong effect on the cellulose DPw. 

### 3.7. Dissolving Ability—NaIO_4_ + Peroxide Treatment

**Figure 8:**
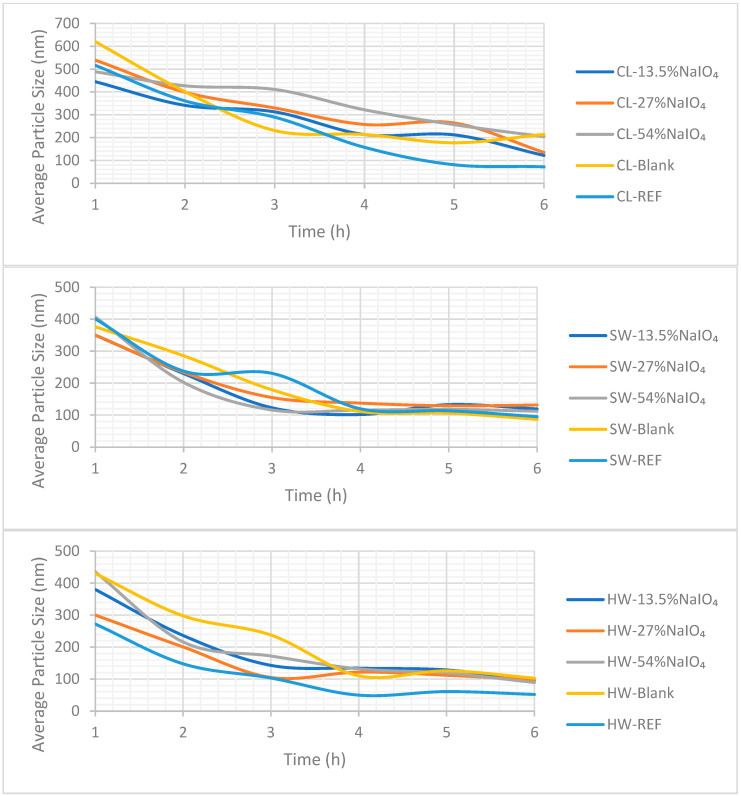
Dissolution ability during carbanilation monitored with DLS for CL, SW, and HW when using different doses of NaIO_4_ prior to H_2_O_2_ oxidation.

Despite the low amount of carboxyl groups introduced into the substrates, potential changes in the dissolving ability were evaluated. Firstly, the results from the DLS are presented in Figure 8; and at the end, the findings from the dissolution in the NaOH:urea:water polar system are discussed.

Concerning CL and SW, as already observed in the previous section, the P stage alone (the so-called “blank trials” without periodate oxidation) did not modify the behavior of the substrates through the carbanilation dissolution test, as the particle size evolution was very similar. The same was observed for the substrates oxidized with NaIO_4_, regardless of the charge applied. This organic dissolution system did not enable the discrimination of the substrates. 

More differences were observed for HW, especially during the first three hours of carbanilation. HW-REF dissolved faster than the oxidized samples. However, compared to the blank trial (P alone), the use of NaIO_4_ accelerated dissolution, yet without dissolving faster than the untreated pulp. It seems that the dissolving ability through carbanilation in DMSO was affected compared to the reference pulp, which may be a consequence of the insertion of COOH groups. The HW-blank presented the most important affectation. Moreover, at the end of the carbanilation reaction, all of the substrates, oxidized or not, were fully dissolved. 

**Figure 9:**
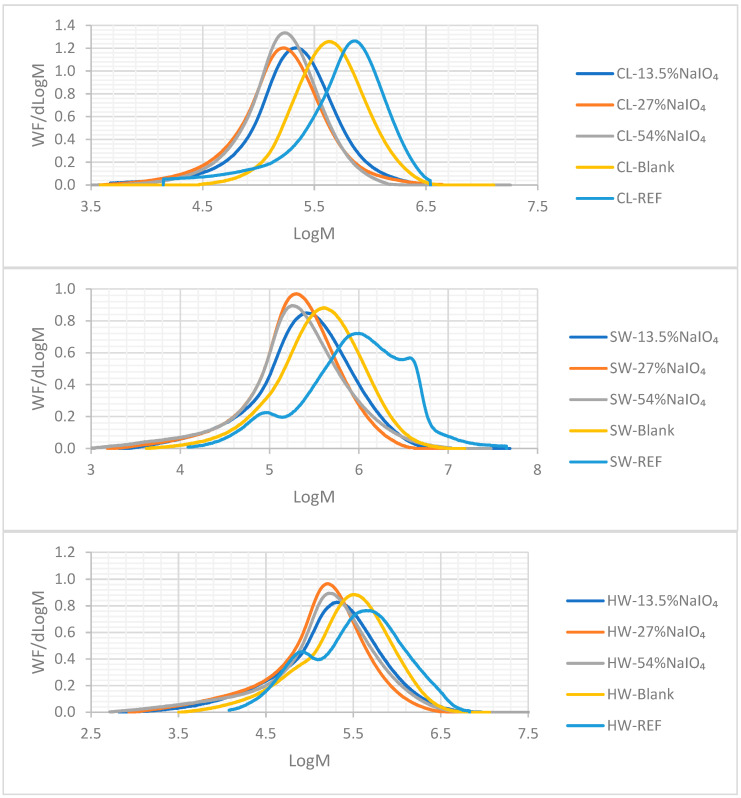
MWD profiles of CL, SW, and HW using different doses of NaIO_4_ prior to H_2_O_2_ oxidation.

After the carbanilation reaction, the MWD profiles were obtained via HPSEC analyses, and these profiles are presented in Figure 9. Additional information extracted from the SEC analyses is reported in Table 6.

The reference profiles for the Kraft pulps (SW and HW) displayed a bimodal profile: at a high molecular weight, the cellulose fraction was present, and at a lower mass, the hemicelluloses fraction was observed, as displayed in Figure 8. For both Kraft pulps, the peroxide oxidation shifted the signal towards lower weights, but a small fraction of hemicelluloses seemed to be still present for the SW-blank. The extent of the degradation is confirmed in the decrease in the DPw values presented in Table 6. As observed earlier in this paper, the addition of NaIO_4_ oxidation prior to H_2_O_2_ oxidation made the shift of the MWD profiles towards lower log M values more prominent. 

Regarding the blank trials for SW and HW using oxidations with peroxide alone, the degradation was lessened as the MWD profile shifted slightly towards a lower mass; but, two fractions were still observed, with the hemicelluloses having a light shoulder in the region of hemicelluloses, as is the effect of a classical P stage, where the substrates can be partially depolymerized without a major impact on the hemicelluloses. The effect of the dose of NaIO_4_ on hemicelluloses removal is more difficult to observe; the signal attributed to hemicelluloses seems to be absent, but due to the strong depolymerization, no clear conclusion can be drawn. What we observed is that the lowest dose of periodate, i.e., 13.5%, leads to a lower depolymerization, whereas using 27% or 54% lead to pulps with similar MWD profiles, leaning towards lower log M values. Another noticeable feature is that the peak of aggregates in the SW-REF disappeared after oxidation, clearly showing that even P oxidation improves the accessibility/reactivity of the substrate.

CL-REF displayed one peak only, evidencing the absence of hemicelluloses (as expected). The results obtained for SW and HW confirms those obtained for the CL: the NaIO_4_ oxidation followed by H_2_O_2_ oxidation was more aggressive for the carbohydrates than the peroxide oxidation alone (blank trial), but the dose of periodate did not induce any significant changes in the MWD profiles (except when using 13.5% NaIO_4_, where degradation was attenuated). Nevertheless, to produce dissolving-grade pulp of a high quality, the depolymerization should be controlled. Thus, the dosage that produced the more interesting results should be selected, i.e. the lower dose, of 13.5% NaIO_4_.

The complementary data from the HPSEC analyses presented in Table 6 confirm the previous discussion about the MWD profiles. In general terms, the DPw of the three substrates was strongly affected by the trials, including the NaIO_4_ oxidations, more than if only a P oxidation was used (as in the blank trials). It was previously stated that the sample with the more interesting viscose-grade characteristics was the blank sample, which also presents a narrower profile with less degradation than the others. A narrow MWD is preferred for dissolving pulp applications to ensure a more homogeneous final product. Unfortunately, when NaIO_4_ oxidation was included, the dispersity increased. Other interesting information obtained in between the oxidations steps is presented in Appendix A. 

### 3.8. Cellulose Solubility in NaOH:Urea:Water System

**Figure 10:**
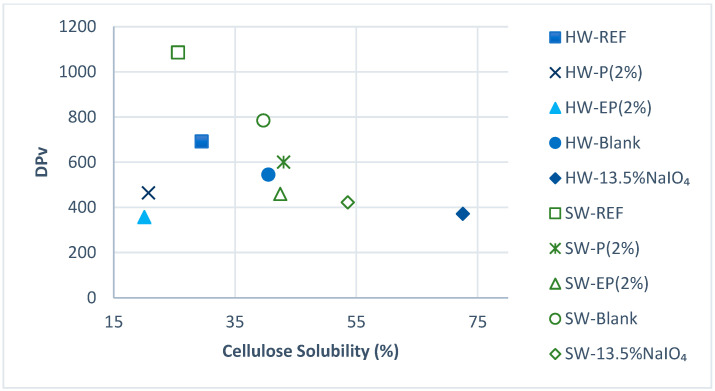
Cellulose solubility (%) in the NaOH:urea:water system of the more interesting treatments for SW and HW.

As our objective was to study the effect of introducing carboxyl groups in the substrates to improve their dissolution behavior, while controlling the substrate depolymerization to reach acceptable dissolving-grade pulp properties, the dissolving ability has also been tested in the NaOH:urea:water system, and these results presented in Figure 10. 

As observed in previous studies, the pulp dissolution in this system is highly correlated with the DPv and is also correlated with the intrinsic viscosity in CuED. Regardless of the substrate origin or treatment, except for HW-EP (2%) and HW-P (2%), the lower the DPv, the better the cellulose dissolution. The non-compliance of HW-EP (2%) may be due to the cellulose II that was formed, thus limiting the dissolution, despite the strong reduction of the DPv in this pulp. In the case of HW-P (2%), the formation of cellulose II could not be invoked, but this pulp was rather strongly oxidized, as seen by the decreased IV in Table 6.

Introducing NaIO_4_ oxidation led to a much-improved dissolution of both the Kraft pulps in this system. This is evidenced when comparing the REF, P, or EP samples to the NaIO_4_ sample, even at the lowest dosage of 13.5%.

The highest improvement can be observed for HW, which, at DPv (CuED) values close to 400, presented a solubility two to three times higher after periodate treatment than that of the HW-blank, HW-P (2%), or HW-EP (2%). SW had the highest initial DPv and its solubility reached about 55% after periodate oxidation, a doubled value compared to the reference (25%). To conclude, the insertion of a periodate oxidation stage prior to peroxide oxidation led to a major solubility improvement in the NaOH:urea:water system, for both HW and SW. 

## 4. Discussion

Two combinations of treatments using hydrogen peroxide as the main oxidant were studied to convert paper-grade bleached pulp to dissolving-grade pulp. 

In the first section, a strong alkaline extraction step was applied prior to hydrogen peroxide oxidation. As a result, more-uniform molecular weight distributions were obtained than with performing the extraction alone (E treatment). With the addition of the hydrogen peroxide oxidation, EP, a higher polymer dispersity was observed, which translates to a broader MWD compared to E alone. E and EP combinations were also able to remove a large part of the hemicelluloses, whereas P alone did not. The extraction of the hemicelluloses seemed easier for HW, which, after EP, reached 96.9% cellulose. For the softwood pulp after EP, cellulose purity reached 90%. These different behaviors could be related to the different type of hemicelluloses present in SW. Despite the interesting results in terms of brightness (>91%) and cellulose purity (>90%) achieved with the EP treatment, the intrinsic viscosity values were below the target needed for a dissolving-grade pulp for viscose applications (400–600 mL/g). Moreover, the cellulose solubility in the NaOH:urea:water system decreased for HW, whereas the SW resulted in a 100% improvement. No significant improvements were observed during cellulose derivatization. As a conclusion, EP treatment was not sufficient to convert bleached Kraft pulp into dissolving-grade pulp.

Our second strategy investigated the use of periodate oxidation prior to hydrogen peroxide oxidation to depolymerize the substrate, and to enrich the pulp with functional groups that may enhance its reactivity. The depolymerization begins with periodate oxidation and is accentuated by the hydrogen peroxide treatment. Intrinsic viscosity values higher than 300 mL/g were difficult to reach, whereas an interesting cellulose solubility in the NaOH:urea:water system was observed, i.e., a solubility which was more than double that of the reference. A slightly slower dissolution during the carbanilation reaction was also observed, which may be an indication of the insertion of functional groups probably affecting the dissolution (carbanilation) in DMSO. 

The conversion of fully bleached Kraft paper-grade pulps into dissolving-grade pulps using hydrogen peroxide as the main chemical was a challenge. Removal of hemicelluloses using alkaline extraction reinforced by H₂O₂ oxidation was not sufficient to improve their dissolving ability, and the oxidative treatments which aimed to insert COOH groups in the pulp damaged the cellulose too much, leading to very low intrinsic viscosity values, off the target. Hence, another method of valorization is proposed: employing these interesting oxidation conditions in order to produce microfibrillated cellulose (MFC). An investigation of the potential uses of the proposed treatments to produce MFC with chemicals commonly found in paper mills has already been published [27].

## 5. Conclusions

The periodate–peroxide system is the most promising of those discussed in this paper since only low amounts of COOH seem to be required to improve the cellulose dissolution in an aqueous solvent. This is very interesting as derivatization and the usage of harmful chemicals could be avoided, and aqueous solvents are cheap and environmentally friendly. The results obtained with the combination of periodate and peroxide oxidation open the door for the development of new cellulose regeneration systems. Another way to optimize this research would be to use Kraft pulp with a higher DPv, since in the present work, the initial substrates contained cellulose with an already low DPv. 

## Figures and Tables

**Table 1:** Operating conditions during the P stage [3].

Conditions	Classical P Stage	(PO) or (OP) Stage
Pressure (bars)	1	3–8
Pulp cons. (%)	10–15	10–35
Temp. (°C)	60–90	80–120
Time (min)	30–240	30–180
NaOH (% odp)	0.2–4	0.2–4
pH	10–12	10–12
H_2_O_2_ (% odp)	0.2–4	0.2–4

Note: odp stands for oven-dried pulp.

**Table 2:** Operating conditions of EP, E, P, and E + P treatments.

Step	Conditions for HW-K-2 and SW-K-1
E	60 min, 75 °C, 75 g NaOH/L
P	60 min, 75 °C, NaOH = 2%, H_2_O_2_ = 10%
E + P	E step + washing until neutral pH + P step
EP	60 min, 75 °C, 75 g NaOH/L, and 10% H_2_O_2_

**Table 3:** Alkali resistance, R18 (%), and COOH quantification values for the samples oxidized with 2% and 5% NaOH.

Treatment	Alkali Resistance, R18 (%)	COOH Quantification (mEq COOH/100 g Dry Pulp)
HW-REF	90.6	7.0
HW-E	99.1	5.6
HW-P (2%)	91.3	7.7
HW-P (5%)	90.7	7.9
HW-EP (2%)	96.9	4.3
HW-EP (5%)	95.3	5.9
SW-REF	79.7	5.9
SW-E	92.2	4.7
SW-P (2%)	84.5	5.2
SW-P (5%)	84.3	5.1
SW-EP (2%)	90.3	5.1
SW-EP (5%)	90.8	4.8

**Table 4:** COOH quantification (meq COOH for 100 g of dry pulp) and alkali resistance (%) after a combination of NaIO_4_ and H_2_O_2_ (13.5% NaIO_4_ on pulp during the periodate oxidation).

Sample	meq COOH/100 g Dry Pulp	R18 Test (%)
CL-REF	3.8	94.9
CL-NaIO_4_	4.7	96.9
CL-Blank	3.5	96.7
SW-REF	4.3	79.7
SW-NaIO_4_	4.5	84.3
SW-Blank	4.4	85.5
HW-REF	6.3	90.6
HW-NaIO_4_	7.9	90.8
HW-Blank	6.7	94.9

**Table 5:** Additional SEC information—substrates treated with E, P, and EP (2% H_2_O_2_).

	IV(dL/g)	Rh(nm)	Mw/Mn	DPw
HW-REF	2.8	27.8	4.1	1249
HW-E	2.4	23.9	2.3	873
HW-P (2%)	2.1	21.6	4.1	814
HW-EP (2%)	2	18	3	635
SW-REF	6.4	51.7	6.3	4025
SW-E	2.9	28.0	2.6	1222
SW-P (2%)	2.4	23.9	5.0	1066
SW-EP (2%)	2	21	2	742

**Table 6:** Additional SEC information—different doses of NaIO_4_ prior to H_2_O_2_ oxidation.

	IV (dL/g)	Rh(nm)	Mw/Mn	DPw
CL-13.5%NaIO₄	1.7	18.2	2.5	569
CL-27%NaIO₄	1.3	15.6	2.5	470
CL-54%NaIO₄	1.4	16.0	1.9	424
CL-Blank	2.9	27.8	1.8	1072
CL-REF	4.2	34.9	2.9	1433
SW-13.5%NaIO₄	2.0	20.9	4.8	883
SW-27%NaIO₄	1.7	18.6	4.1	644
SW-54%NaIO₄	1.5	18.2	7.3	835
SW-Blank	2.6	25.6	3.7	1205
SW-REF	6.4	51.7	6.3	3699
HW-13.5%NaIO₄	1.6	17.9	7.1	683
HW-27%NaIO₄	1.3	15.1	5.8	456
HW-54%NaIO₄	1.3	16.3	9.3	588
HW-Blank	2.2	22.4	4.1	862
HW-REF	2.8	27.8	4.1	1215

**Figure A1:**
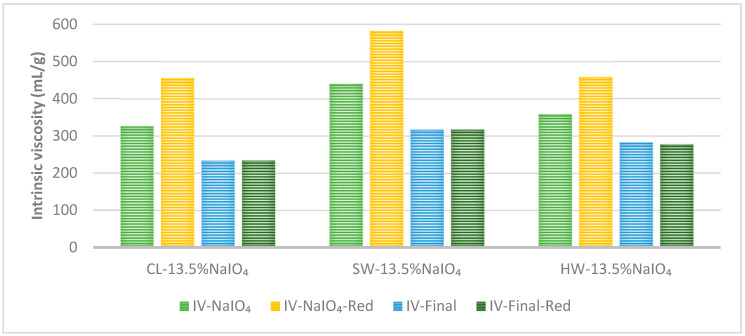
Intrinsic viscosity (mL/g) measured with and without NaBH_4_ reduction for 13.5%NaIO_4_.

**Figure A2:**
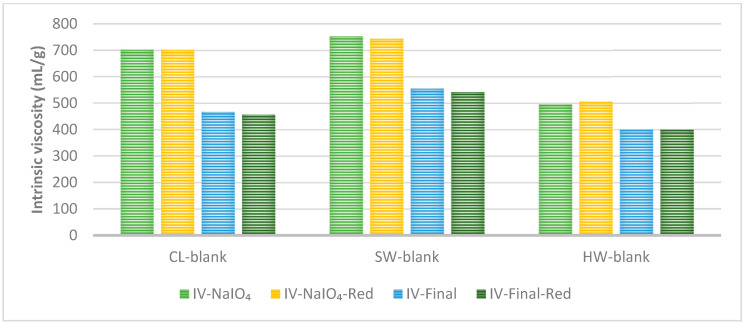
Intrinsic viscosity (mL/g) measured with and without NaBH_4_ reduction for the blank trials.

## Data Availability

The data presented in this study are available on request from the corresponding author.

## Appendix A

The intrinsic viscosity was also measured after the first oxidation, with or without NaBH_4_ reduction, prior to the dissolution in CuED. Results for the 13.5% NaIO_4_ are presented in Figure A1, and results for the blank in Figure A2.

It was confirmed, for all of the three substrates, that the IV values obtained after periodate oxidation without the reduction with NaBH_4_ were significantly lower than after NBH_4_ reduction (with more than 30% difference, which evidences the formation of carbonyl groups during the first oxidation). Even after NaBH_4_ reduction, the IV values of the periodate oxidized substrates were lower than those of the reference substrates. This shows that periodate is also responsible for cellulose depolymerization. The values obtained with the blank trial confirm the reliability of this method: as no NaIO_4_ was used in the blank trail, no carbonyl was formed, and there was no change in IV compared to the reference.

During the second oxidation with H_2_O_2_, the depolymerization continued, as lower IV values were observed compared to the values seen after the first oxidation. However, the values obtained after the second oxidation were similar with and without the reduction with NaBH_4_. These results confirm that the oxidation with H_2_O_2_ in alkaline conditions at 75 °C converted all of the carbonyls created in the periodate oxidation into carboxyls. No changes were observed for the blank trials, with or without NaBH_4_ reduction, as expected.
